# Metformin effectively alleviates the symptoms of Alzheimer in rats by lowering amyloid β deposition and enhancing the insulin signal

**DOI:** 10.1007/s11011-024-01422-8

**Published:** 2024-11-25

**Authors:** Hamed A. Abosharaf, Yasmin Elsonbaty, Ehab Tousson, Tarek M. Mohamed

**Affiliations:** 1 Biochemistry Division, Chemistry Department, Faculty of Science, Tanta University, Tanta, 31527 Egypt; 2 Zoology Department, Faculty of Science, Tanta University, Tanta, 31527 Egypt

**Keywords:** Alzheimer’s disease, Amyloid β, Insulin resistance, Metformin, Glucose impairment

## Abstract

Alzheimer’s disease (AD) exhibits distinct biochemical and histopathological attributes, encompassing cellular, neuronal, and oxidative impairment. There is also an abnormal buildup, misfolding and clumping of amyloid β (Aβ). The present study aimed to explore the influence of the antihyperglycemic agent metformin on rats with AD-like symptoms, while also elucidating the intricate relationship between insulin resistance and AD. The rats were categorized into five groups: a control group, a saline-administered group, a metformin-treated group, AD-model rats, and AD-rats treated with a 200 mg/kg dose of metformin. Cognitive impairment was rated using the classical labyrinth test. Moreover, serum biochemical parameters, encompassing glucose levels, Homeostatic Model Assessment for Insulin Resistance (HOMA-IR), Glycated hemoglobin (HbA1c), lipid profile, kidney, and liver function, were evaluated. Additionally, oxidative, antioxidant, and neurotransmitter parameters were measured in hippocampus tissues. Also, the Aβ and insulin receptor substrate 2 (IRS-2) were measured by immunoblotting. Besides hippocampal histopathology, glial fibrillary acidic protein (GFAP) and calretinin immunoreactivity were monitored. The study findings disclosed deficits in memory and learning capabilities among AD rats. Furthermore, AD-afflicted rats exhibited heightened glucose levels, elevated HOMA-IR and HbA1c values, alongside compromised liver, and kidney functions. Additionally, an upsurge in oxidative stress coincided with a notable reduction in the antioxidant system and neurotransmitters activities. The levels of Aβ deposition increased, while IRS-2 expression subsided, accompanied by alterations in the hippocampal structure and neuronal damage. These changes were paralleled by an intensification in GFAP reactivity and a detracting in calretinin reactivity. Metformin was altogether able to move forward cognitive execution by means of bringing down oxidative stress and Aβ conglomeration. Furthermore, metformin was able to improve neurotransmitters and insulin signals. AD, glucose impairment, and brain insulin resistance are completely interlinked, and future AD medications may be inspired by diabetic medication.

**Supplementary Information:**

The online version contains supplementary material available at 10.1007/s11011-024-01422-8.

## Introduction

From both a biomedical and neurological perspective, Alzheimer’s disease (AD) stands as the foremost cause of dementia, characterized by the presence of amyloid β (Aβ) plaques and tau neurofibril tangles (Apter et al. 2015; Knopman et al. 2021; Uwishema et al. 2022). While AD is conspicuously coupled with amnestic cognitive symptoms, there are erratic instances where non-amnestic symptoms manifest as well (Knopman et al. 2021). AD patients may experience a spectrum of cognitive decline, from mild memory impairment to severe difficulties with daily tasks (Jessen et al. 2014).

Historically, AD was deemed to have a clinical origin, yet it is now unequivocally recognized as being rooted in neurological factors. The etiology of AD is intricately intertwined with various elements, notably metabolic disorders such as diabetes mellitus (Petersen 2018**)**. In the incipient stages of AD, disturbances in glucose with decreased brain glucose utilization have been pinpointed. This may elucidate the two-way liaison between brain insulin resistance and AD (Li et al. 2016; Shinohara and Sato 2017; Pilipenko et al. 2020). Additionally, it has been reported that compromised brain glucose turnover leads to neuroinflammation, further triggering neurodegenerative symptoms by disrupting synaptic plasticity, neuronal viability, and intercellular communication (Zhang et al. 2018).

Despite the availability of acetylcholinesterase inhibitors, effective treatments for AD remain notably lacking (Rygiel 2016). In fact, no new medications have obtained approval from regulatory agencies such as the Food and Drug Administration (FDA) or the European Medicine Agency (EMA) (Apter et al. 2015). Given the outlook for AD and its connection to diabetes, there is considerable interest in antidiabetic medications as potential innovative therapies for AD.

The approved drug metformin, belonging to the class of biguanides and renowned for its antihyperglycemic effects, is currently a cornerstone in the management of type 2 diabetes mellitus (T2DM) (Ferrannini 2014). Previous investigations have sought to elucidate the antihyperglycemic properties of metformin. One study suggested that metformin effectively reduces blood glucose levels without inducing noteworthy hypoglycemia, achieved through its ability to lessening intestinal glucose absorption, augment peripheral glucose uptake, and worsen the insulin sensitivity (Grzybowska et al. 2011). Another inquiry highlighted metformin’s capacity to activate AMP-activated protein kinase (AMPK), subsequently inhibiting gluconeogenesis (Matthaei and Greten 1991). Notably, owing to its minimal side effects, metformin has been explored for various other conditions, including cancer (Coyle et al. 2016), inflammation (Liu et al. 2023), cardiovascular disease (Benjamin et al. 2017), osteoarthritis (Lambova 2023), and dementia (Kumar et al. 2023) In light of the proposed connection between neurodegenerative and metabolic disorders, particularly insulin resistance, our study, along with others, postulates that metformin could offer a novel mechanism of action in these conditions (Beeri et al. 2008; Sonnen et al. 2009). This study seeks to explore the potential of metformin against the symptoms of Alzheimer’s disease induced in rats.

## Materials and methods

### Animals

The rats employed in this study were managed and cared for following the research animal guidelines of the Faculty of Science, Tanta University, Egypt, which adheres to the AARIVE guidelines (Kilkenny et al. 2010) (Official ethical No. IACUC-SCI-TU-0259). Fifty male Wistar rats, weighing between 200 and 220 g and aged 6–7 weeks, were procured from the animal and breeding unit at VACSERA in Cairo, Egypt. This age of mice is useful for behavior studies since, up to five months of age, there was a natural decline in behavioral activity, motor activity, and several rearing rats (Sudakov et al. 2021). Additionally, female mice were not employed in this investigation to eliminate any variability related to the estrous cycle (Deacon 2006). The rats were maintained at a temperature of 25 °C under a regular day-night cycle and were given free access to food and water. A minimum acclimatization period of one week was provided before commencing the experiment (Kilkenny et al. 2010; Percie du Sert et al. 2020).

## Experimental section

### Alzheimer’s disease initiation in rats

Five distinct groups were established, each comprising 10 rats per cage that were randomly selected. Further, blinding is not included in the current study due to measuring overt mental activity using videos. G1(Normal): represented untreated rats. G2 (Saline): consisted of control rats receiving saline injections throughout the experiment. G3 (Metformin): included control rats treated with a continuous dose of 200 mg/kg metformin (Sigma-Aldrich, USA). G4 (AD model): comprised AD-induced rats that were orally administered 120 mg/kg D-galactose (D-gal) Sigma-Aldrich, USA) and 50 mg/kg of aluminum chloride (AlCl_3_) (ACROS organics, Geel, Belgium) daily for a month, (Mallikarjuna et al. 2016). G5 (AD + Metformin): involved AD-rats initially subjected to the conditions of G4 and subsequently treated with an oral dose of 200 mg/kg metformin daily for a month (Zhang et al. 2017). Changes in body mass were recorded, and cognitive impairment, a hallmark of AD, was assessed using the Classic Labyrinth method (Gasmi 2018; Abosharaf et al. 2024). At the experiment’s conclusion, the rats were euthanized with an intraperitoneal injection of pentobarbital (40 mg/kg), followed by cervical dislocation. Following confirmation of rat death, the brains were extracted, weighed, and hippocampal tissues were collected, with a portion preserved for histopathology and the remainder stored at −80 °C for future analysis. The pancreas was harvested, washed with saline buffer, and preserved in 10% formalin for histopathological examination.

## Serum biochemical measurements

Blood samples were collected from the central retinal vein, and serum was obtained by incubating the whole blood at room temperature for 30 min followed by centrifugation at 1100xg for 15 min. The resulting serum (*n* = 10) was used to measure glucose (Cat No. GL13 20), liver function markers (ALT, Cat. No. AL 10 31, and AST, Cat. No. AS 10 61), kidney function markers including urea (Cat. No. UR 21 10) and creatinine (Cat No. CR 12 51), further the levels of cholesterol (Cat. No. CH 12 20), triacylglycerol (Cat. No. TR 20 30), HDL (Cat. No. CH 12 31), LDL (Cat. No. CH 12 31), iron (Cat. No. IR 15 10) and zinc levels (Cat No. Zn 21 20). These measurements were performed according to kit instructions (Bio-diagnostic Inc, Egypt). Additionally, Rat insulin resistance (HOMA-IR) and insulin concentration ELIZA kit was gained from Invitrogen (USA, Cat. No. ERINS).

## Neurochemical markers assessment

Neurotransmitters including dopamine, acetylcholine, and acetylcholine esterase were quantified (*n* = 10) in the hippocampus tissues of all treated rats. Dopamine levels were assessed using a kit from Eagle Bioscience (Cat. No. 50–813-97, Amherst, USA), acetylcholine concentration was determined with a rat acetylcholine kit from My BioSource (Cat. No. MBS728879, San Diego, USA), and acetylcholine esterase activity was measured using a rat acetylcholine esterase (AChE) ELISA kit from Elabscience (Cat No. E-EL-R0355, Texas, USA).

### Quantification of the oxidative/ antioxidative parameters

Oxidative stress was evaluated in the hippocampus of all rats (*n* = 10) by measuring malonaldehyde (MDA) levels, as previously described (Niehaus Jr and Samuelsson 1968) (Detailed method listed in supporting data). Antioxidant factors including glutathione, superoxide dismutase (SOD), glutathione peroxidase (GPx), and catalase (CAT) were determined using appropriate assays (Ellman 1959) (Detailed method listed in supporting data). Superoxide dismutase (SOD) (Cat. No, SD 25 21) was measured following the kit instruction obtained from Bio-diagnostic, Egypt (Nishikimi et al. 1972). Glutathione peroxidase (GPx) (Cat. No. GP 2524) was evaluated using marketable set kit from (Bio-diagnostic, Egypt) (Paglia and Valentine 1967). Furthermore, the catalase activity (CAT) was estimated using kit obtained from Bio-diagnostic, Egypt (Cat. No. CA 25 17) (Aebi 1984; Radwan et al. 2021).

## Immunoblotting analysis

Protein concentration was determined using a Bradford assay Abcam kit (Cat. No. ab102535), and protein was extracted from hippocampus tissues using a specific protein extraction kit set from Abcam (Cat. No. ab270054). Protein samples (20 µg) were separated on 12% SDS-PAGE and transferred to a polyvinylidene difluoride membrane. The membrane was blocked with skimmed milk and incubated with 1:1000 diluted primary antibodies; Aβ (Cat. No. 2454) (Verma et al. 2023) that provided by Cell Signaling, Massachusetts, USA) and IRS-2 (Cat. No. sc-390761, Santa Cruz Biotechnology, Texas, USA) (Chen et al. 2021) followed by secondary antibodies (HPR-goat anti-rabbit IgG (H&L), (ab205718), Abcam, UK). β actin (sc-69879, Sant Cruz) was used as internal control. The specificity of the antibodies was not confirmed in the current study, we based on the supplier company guidelines and appearance single band on the membrane. Protein bands were visualized using chemiluminescence (ECL) and further quantified using ImageJ 1.39P software (National institute of health, USA) (Beverloo et al. 1992; Radwan et al. 2021).

## Histopathological and immunohistochemical investigations

Hippocampus and pancreas tissues were preserved in formaldehyde, processed, and sectioned for histopathological examination using hematoxylin and eosin staining (Tousson et al. 2012). For immunohistology, Hippocampus sections were deparaffinized, hydrated, and treated with hydrogen peroxide blocking solution before incubation with 1:1000 diluted primary antibodies GFAP (Cat. No. No. Z0334) (Günther et al. 2021) and calretinin (Cat. No. No. IR627) (den Braber-Ymker et al. 2017) which were supplied by Agilent Dako (Santa Clara, CA, United States) then incubated with secondary antibody peroxidase conjugated (EnVision kit, Cat. No. K40023, Dako; Agilent). Finally, about three microscopic fields for each sample were recorded for all different samples (*n* = 5) in each experimental group utilizing Olympus microscope (BX41) with digital canon 620 camera (Olympus Corporation). The contrast and resolution were improved using Adobe photoshop package. For quantitative analysis of immunohistology reactions, ImageJ software was utilized to calculate the interaction area related to the total area of the filed (Happerfield et al. 1993; Guinard-Samuel et al. 2009; Amin et al. 2013).

### Statistical assessment

The sample size (n) in the study was determined based on a level of confidence (95%) and population size (*N* = 10) which involved in the study (Supporting information for details). Data analysis was conducted using GraphPad Prism software version 6. One-way ANOVA with Tukey’s multiple comparison test was employed to compare measurable variables between study groups. Data are presented as mean ± SD, and a significance level of *p* ≤ 0.05 was considered statistically significant (Altman 2005**)**. Additionally, the normality and homogeneity of samples were employed using D’Agostino-Pearson omnibus and Shapiro-Wilk tests in GraphPad prism. The Data which is not normally distributed was analyzed by one-way ANOVA (nonparametric) with Bonferroni-Holm correction using GraphPad prism and presented as median with interquartile range.

## Results

### Metformin helps improve memory and learning in rats with Alzheimer’s disease

**Fig. 1:**
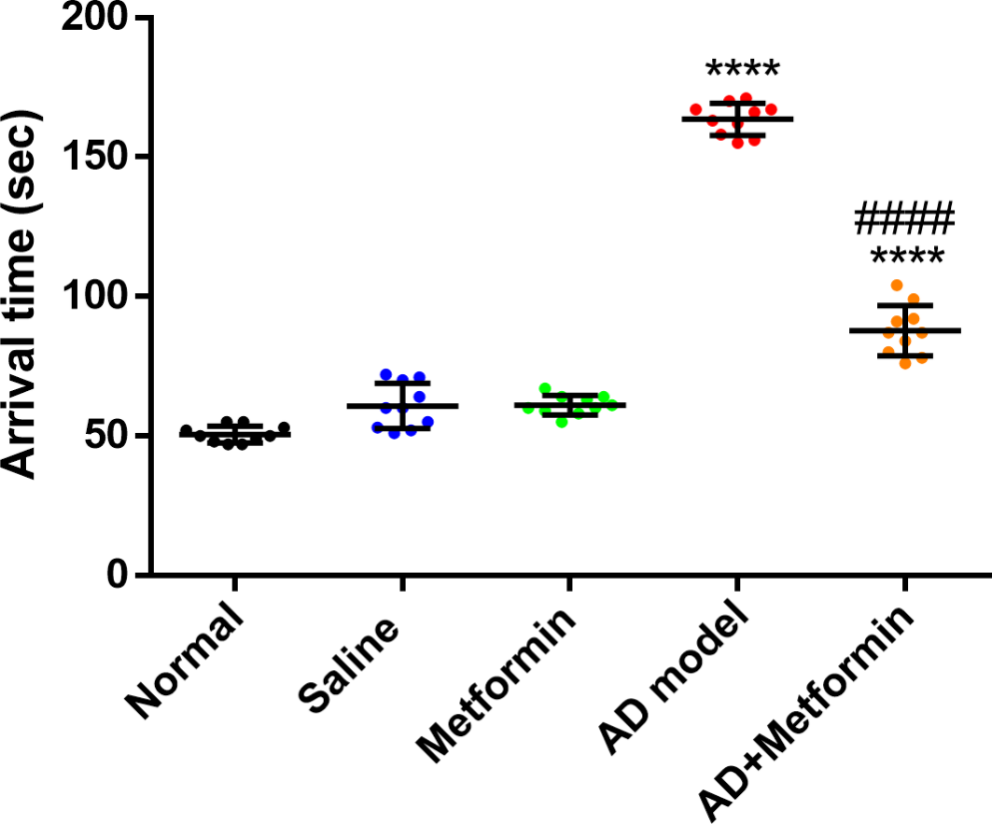
Metformin improves the arrival time using the labyrinth in an AD-induced rat model. The results were presented as mean ± SD, (*n* = 10). where ^****^P < 0.0001 vs. normal group and ^####^*P* < 0.0001 vs. AD-rat model

As depicted in Fig. 1, administration of D-gal and AlCl_3_ led to significant deficits (*p* < 0.0001) in learning and spatial memory in the AD-induced rats (G4) compared to the control group (G1). Furthermore, when compared to the untreated AD-induced rats (G4), treatment with 200 mg/kg of metformin resulted in improved memory with a notable reduction (*p* < 0.0001) in arrival time in the AD-metformin treated group (G5). Notably, no substantial change in arrival time was observed in non-AD groups receiving saline (G2) or metformin (G3) in comparison to the control group (G1). Additionally, there was a clear reduction (*p* < 0.0001) in body weight in the AD rat model compared to controls, which was restored following metformin administration. Moreover, the brain weights did not exhibit meaningful reductions in AD rats when compared to the control group (Fig. S1, supporting data).

### Metformin assists in lowering hyperglycemia, enhancing insulin sensitivity, and mitigating liver and kidney impairments in AD rats

**Fig. 2:**
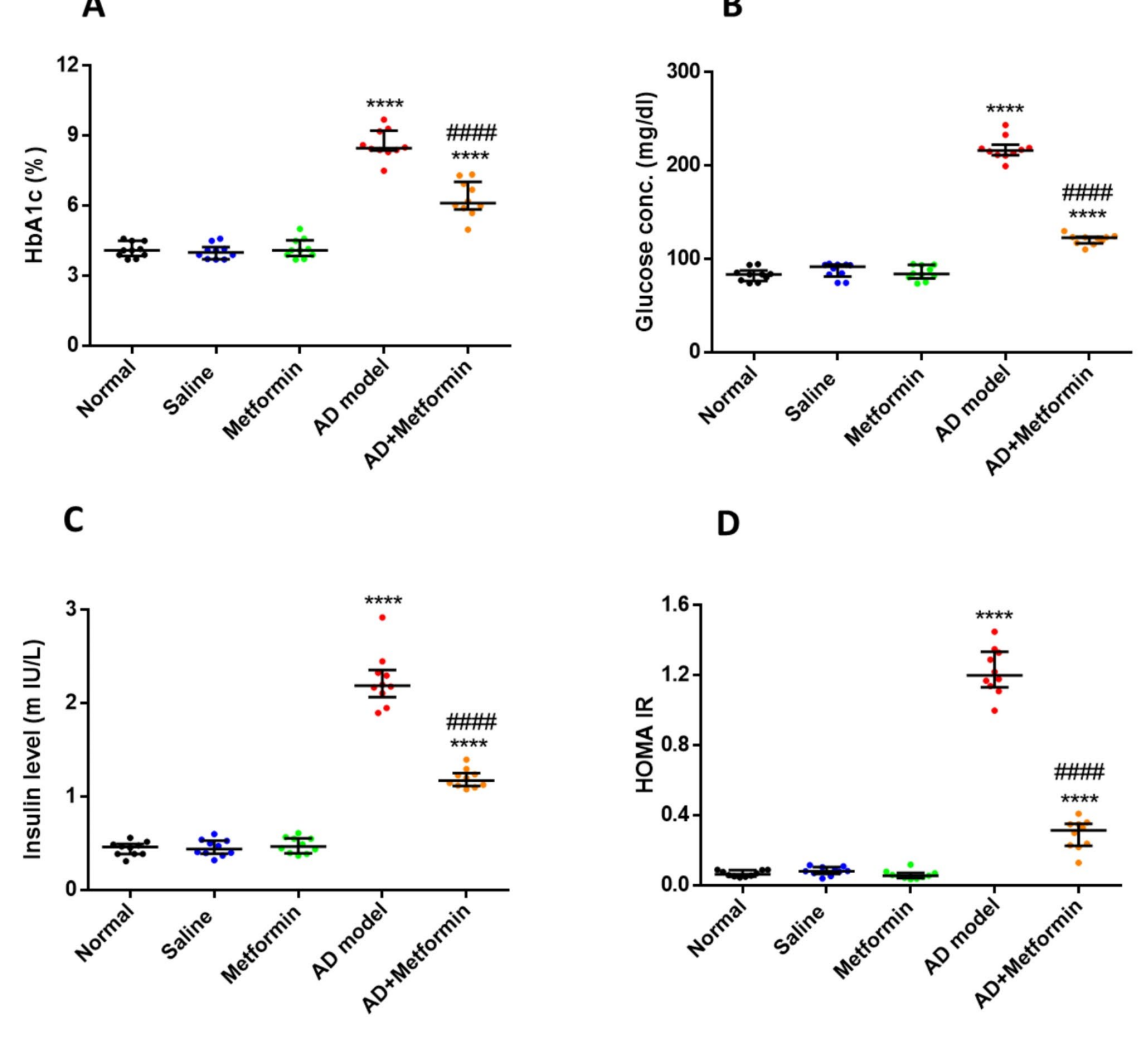
Metformin lowers serum HbA1c, glucose, insulin, and HOMA IR in an AD-induced rat model. (A): The level of glycated hemoglobin in treated rats. (B): The glucose level of the rats involved in the study. (C) the level of insulin in all rats. (D) HOMA IR, insulin resistance marker, in all groups. The results were presented as mean ± SD, (*n* = 10), where ^****^P < 0.0001 vs. normal group and ^####^*P* < 0.0001 vs. AD-rat model

AD is closely linked to metabolic disorders like diabetes and can affect various bodily processes. So, we measured glucose levels, insulin resistance, and the functioning of the kidneys and liver. As shown in Fig. 2, our serum analysis data revealed a substantial increase (*p* < 0.0001) in HbA1c, glucose levels, insulin, and insulin resistance (HOMA-IR) in the AD model group (G4) in contrast to the control group (G1). Furthermore, post-treatment of AD-induced rats (G5) with a 200 mg/kg dose of metformin exhibited a marked reduction (*p* < 0.0001) in the aforementioned biomarkers compared to untreated AD rats (G4). Moreover, AD-induced rats (G4) displayed impaired liver and kidney function along with decreased serum iron and zinc levels, which were subsequently improved after metformin treatment (Table S1, supporting data). Additionally, as summarized in (Table S1, supporting data), the AD rat model demonstrated elevated levels of cholesterol, triacylglycerol, and LDL, accompanied by a notable reduction in HDL.

### Metformin improves neurochemical transmitters in AD rats

**Fig. 3:**
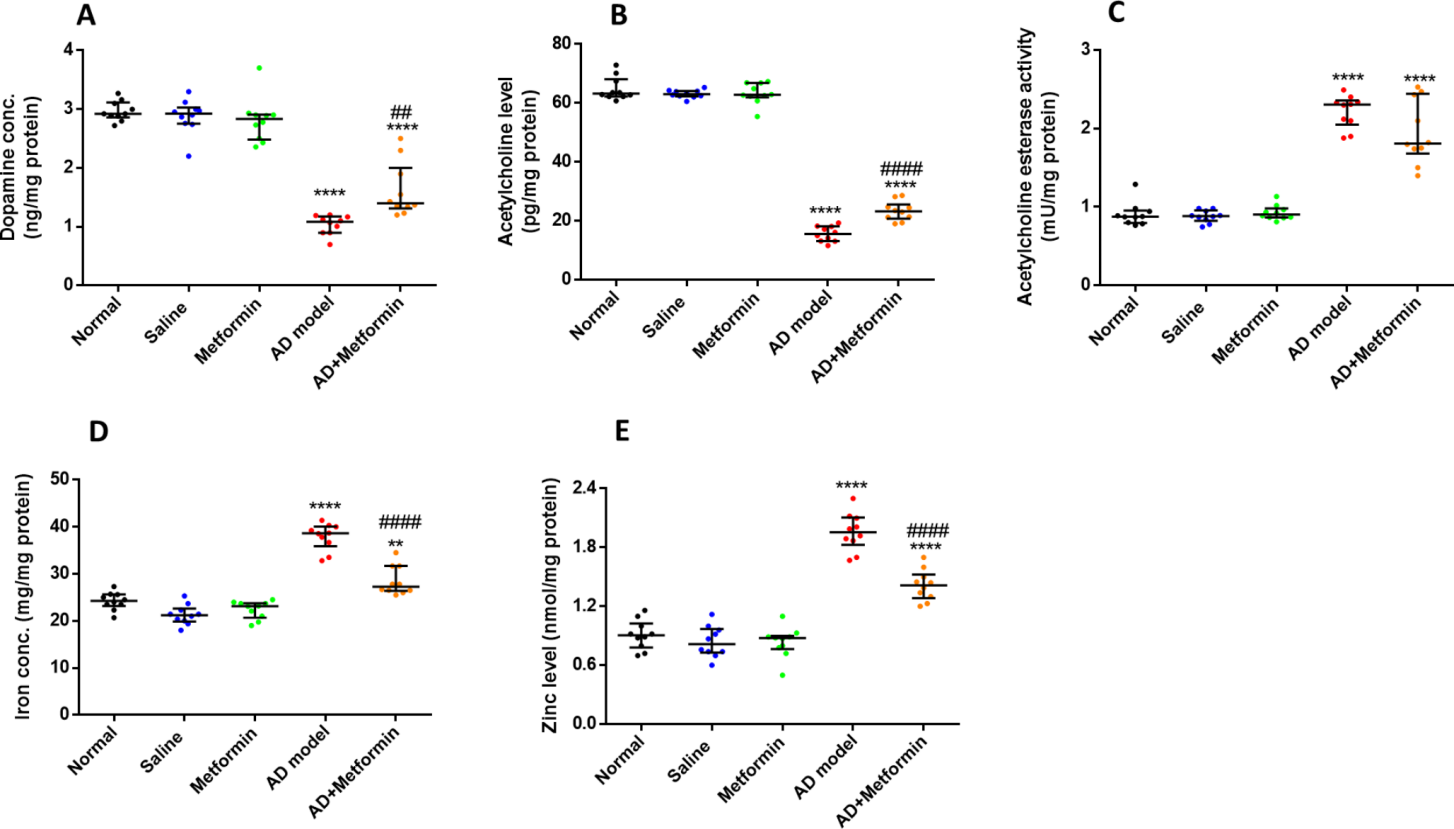
Metformin boosts neurotransmitters in the hippocampus. (A): Hippocampal dopamine level in treated rats. (B): Hippocampal acetylcholine level in all treated rats. (C): activity of acetylcholine esterase in hippocampus tissues in rats involved in this study. (D): iron level in hippocampus tissue. (E): Hippocampal zinc concentration in treated rats. The results were described as median with interquartile range, (*n* = 10), where ^****^P < 0.0001 vs. normal group and ^##^*P* < 0.01, ^####^*P* < 0.0001 vs. AD-rat model

Hippocampal neurotransmitters such as dopamine, acetylcholine, and acetylcholine esterase were measured in the current study as they played a substantial role in AD pathogenesis. The obtained data indicated a pronounced decrease in dopamine (*p* < 0.0001) and acetylcholine (*p* < 0.0001) levels, along with a real increase (*p* < 0.0001) in acetylcholine esterase activity in AD rats compared to control (G1). Importantly, the levels of these neurotransmitters were ameliorated in AD rats treated with metformin (G5) after a four-week administration of 200 mg/kg metformin, as depicted in Fig. 3A and B, and 3C. Additionally, iron (*p* < 0.0001) and zinc (*p* < 0.0001) levels were extensively raised in the AD rat model (G4) compared to the control group (G1). Importantly, iron and zinc levels were dropped after metformin administration (*p* < 0.0001) in AD-post treated rats (G5) (Fig. 3D and E).

### Metformin mitigates oxidative status and enhances the antioxidant system in an AD rat model

**Fig. 4:**
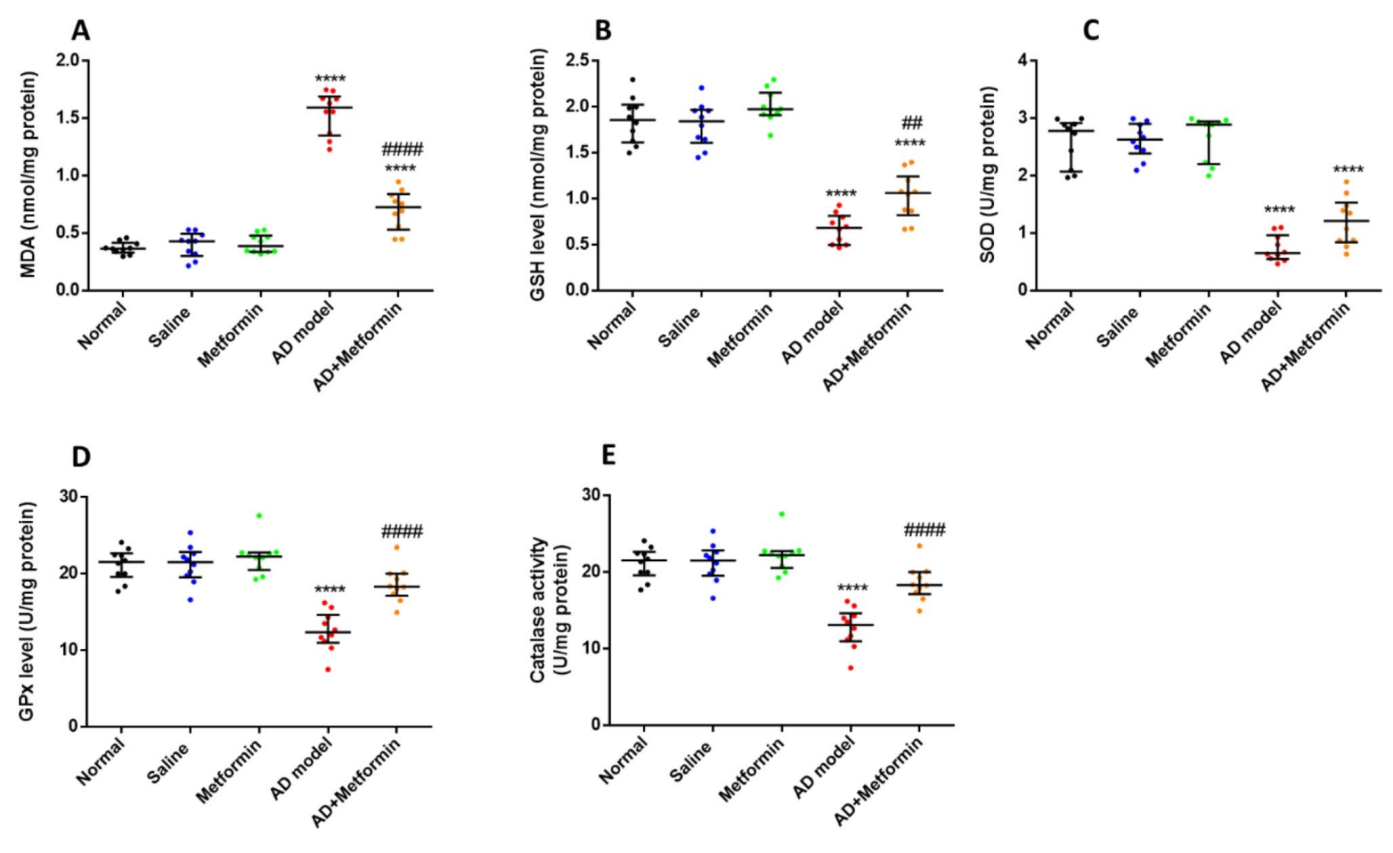
Metformin mitigates oxidative stress and triggers antioxidants in hippocampus. (A): Hippocampal malonaldehyde (MDA) level in treated rats. (B): Hippocampal glutathione (GSH) in all treated rats. (C): The activity of superoxide dismutase (SOD) in hippocampus tissues in rats involved in this study. (D): Glutathione peroxidase activity (GPx) in hippocampus tissues. (E): Hippocampal catalase activity (CAT) in treated rats. The results were presented as median and interquartile range, (*n* = 10), where ^****^P < 0.0001vs normal group and ^##^*P* < 0.01, ^####^*P* < 0.0001 vs. AD-rat model

The progression of AD is exacerbated by the well-established involvement of oxidative stress. ANOVA analysis of hippocampal oxidative status revealed a substantial increase (*p* < 0.0001) in hippocampal MDA, a marker of oxidative stress, in AD rats (G4) compared to the control group. Treatment with 200 mg/kg of metformin was associated with a large drop (*p* < 0.0001) in hippocampal MDA levels compared to the AD model (G4) (Fig. 4A). Additionally, the increased MDA levels in AD rats were accompanied by decreased levels of all antioxidant markers: glutathione (GSH) (*p* < 0.0001), superoxide dismutase (SOD) (*p* < 0.0001), glutathione peroxidase (GPx) (*p* < 0.0001), and catalase (*p* < 0.0001) compared to the control group (G1). Administration of metformin pointedly enhanced antioxidant markers GSH (*p* = 0.0081), SOD (*p* = 0.0448), GPx (*p* < 0.0001), and catalase (*p* < 0.0001) in AD rats (G4) as shown in Fig. 4B, C and D, and 4E, respectively.

### Metformin lowers hippocampal Aβ deposition and insulin resistance

**Fig. 5:**
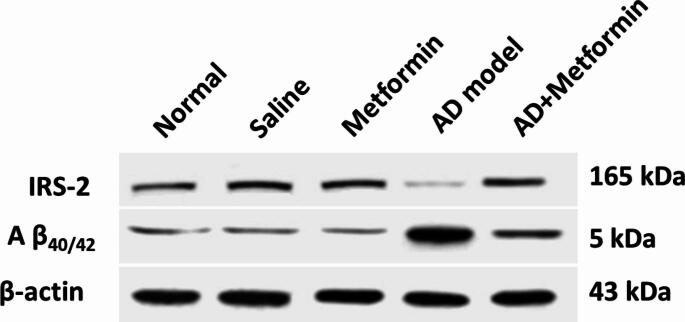
Influence of metformin on amyloid β aggregation (Aβ_40/42_) and insulin receptor substrate-2 (IRS-2) in AD- induced rats and after they received 200 mg/kg of metformin

The impact of metformin on the formation of Aβ plaques, a well-known marker of AD, was explored through western blot analysis. Immunoblotting revealed an increase in Aβ deposition in the AD model (G4), which were reduced following metformin administration in the AD + Metformin group (G5). To assess insulin resistance in the brains of AD rats, insulin receptor substrate 2 (IRS-2) levels were measured, revealing a decrease compared to controls. Importantly, metformin treatment ameliorated this decline in the insulin signaling pathway (Fig. 5).

### Metformin alleviates histopathological alterations in the AD model

**Fig. 6:**
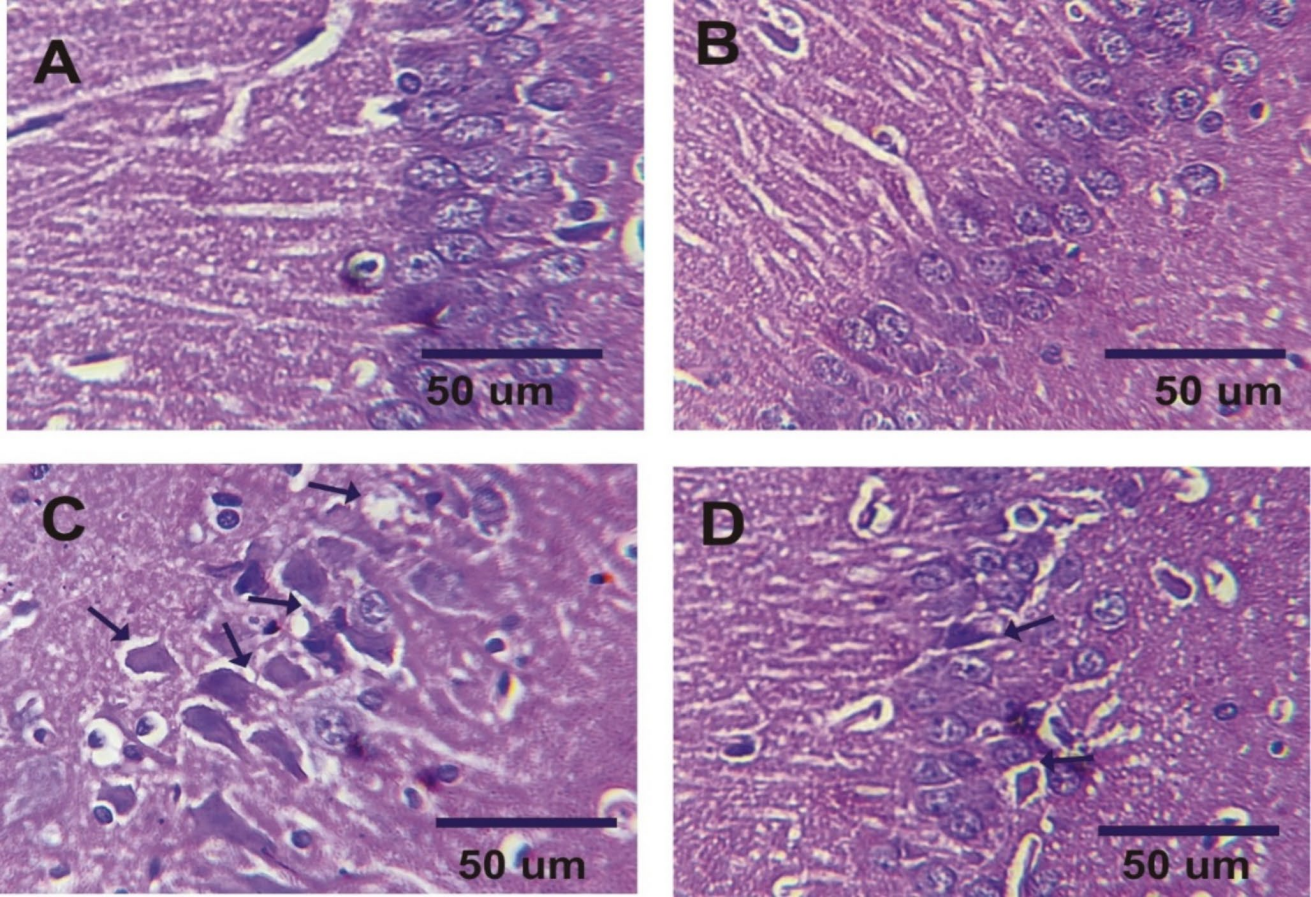
Hematoxylin and eosin-stained hippocampal sections of all treated rats. (**A**): normal rats. (**B**): rats received 200 mg/kg of metformin. (**C**): AD-induced rats. (**D**): AD-rats treated with 200 mg/kg of metformin for one month

**Fig. 7:**
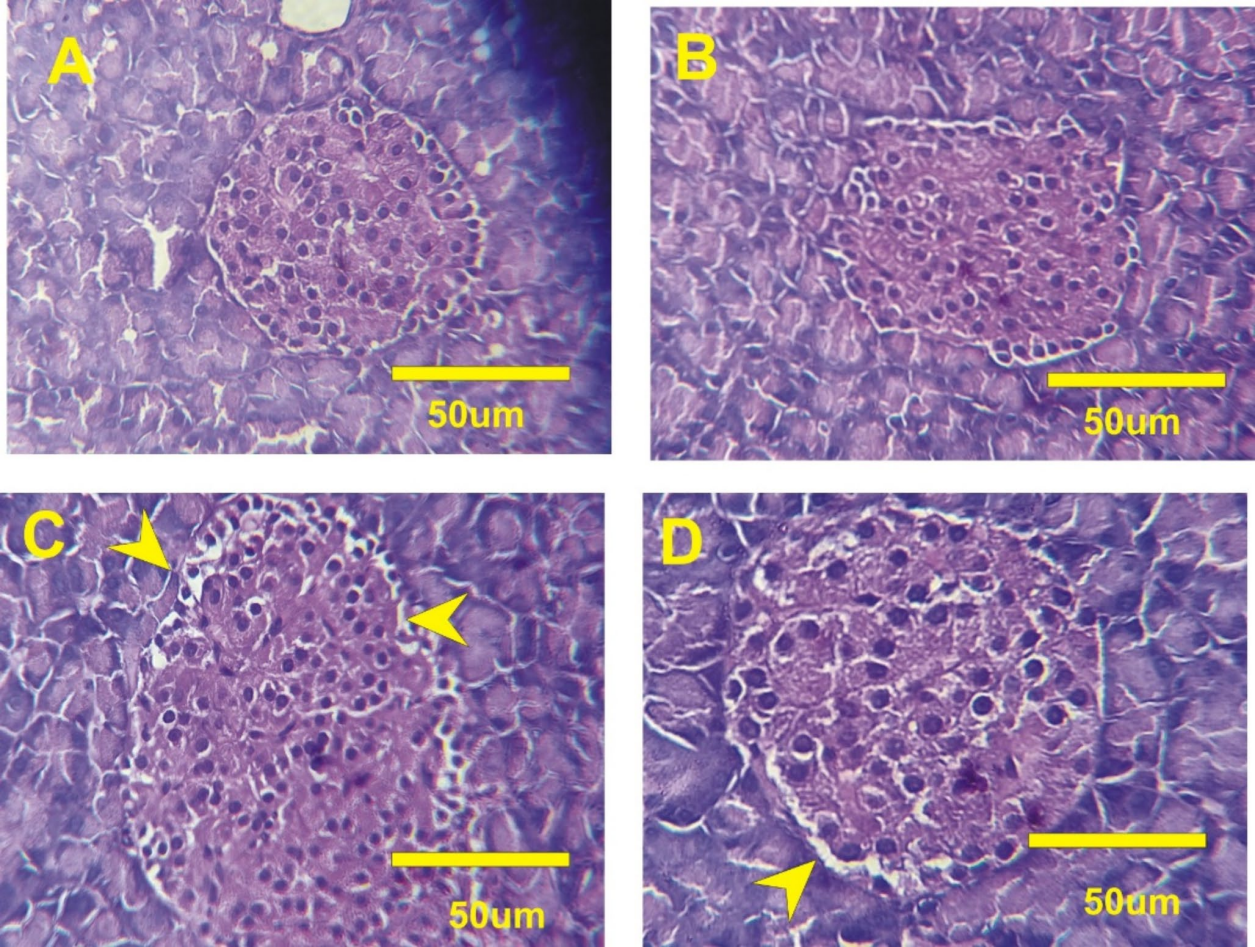
Pancreatic sections of all treated rats. (**A**): normal rats. (**B**): rats received 200 mg/kg of metformin. (**C**): AD-induced rats. (**D**): AD-rats treated with 200 mg/kg of metformin for one month

AD is accompanied by structural changes such as neurons deprivation and neurofibrillary tangles. Hence, histological investigation is used as diagnostic machine for AD. Hippocampal sections of control rats (normal and metformin-treated) displayed well-organized and compact pyramidal cells with a normal molecular layer (Fig. 6A and B). In contrast, the hippocampus of AD-induced rats exhibited pyramidal cell disorganization and vacuolation, along with enlarged neurons and glial cells in the molecular layer (Fig. 6C). Treatment with metformin led to enhanced pyramidal cell organization, reduced vacuolation, and relatively normal cells in the molecular layer (Fig. 6D). Additionally, photomicrographs of pancreatic tissue from control rats (normal and metformin-treated) revealed typical rounded Langerhans islets with normal β-cell distribution (Fig. 7A and B). In the AD rat model, numerous vacuoles and overall distortion of Langerhans islet shape were observed (Fig. 7C). Metformin treatment restored the normal shape of Langerhans islets and reduced vacuole formation (Fig. 7D).

### Metformin lessens the immunoreactivity of GFAP and enhances calretinin in AD rats

**Fig. 8:**
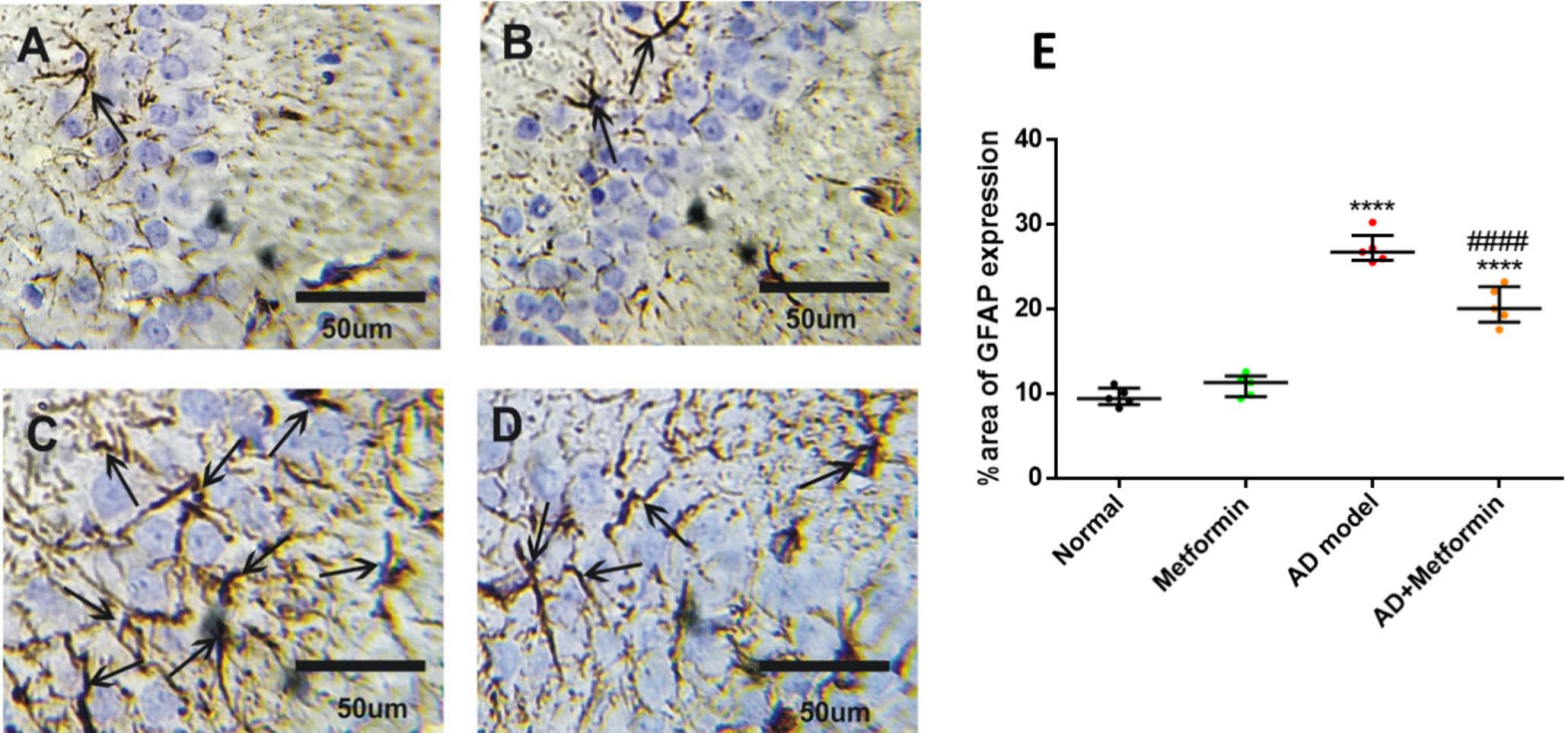
Hippocampal GFAP immunoreactivity. (**A**): normal rats. (**B**): normal rats received metformin. (**C**): AD-rat model. (**D**): AD-rats treated with 200 mg/kg of metformin. (**E**): representative histography of %area of GFAP expression analyzed utilizing ImageJ (*n* = 5). where ^****^P < 0.0001 vs. normal and ^####^*P* < 0.0001 vs. AD model

**Fig. 9:**
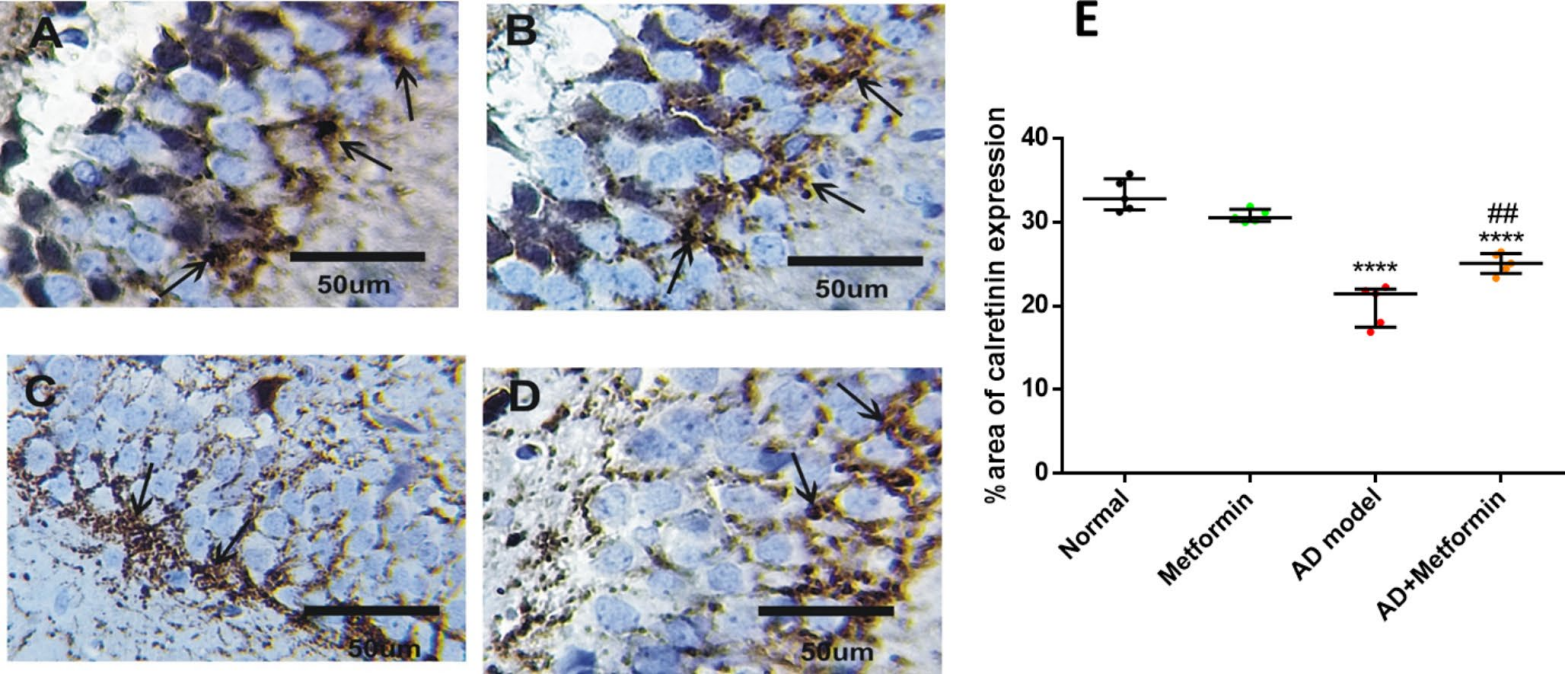
Hippocampal calretinin immunoreactivity. (**A**): normal rats. (**B**): normal rats received metformin. (**C**): AD-rat model. (**D**): AD-rats treated with 200 mg/kg of metformin. (**E**): representative histography of %area of calretinin immunoreactivity analyzed utilizing ImageJ (*n* = 5). where ^****^P < 0.0001 vs. normal and ^##^*P* < 0.01, ^####^*P* < 0.0001 vs. AD model

GFAP and calretinin, modulators of interneuron, were reported to be linked to Aβ aggregation. Immunostaining of GFAP in the hippocampus of normal and metformin-treated groups displayed minimal reaction and normal spreading (Fig. 8A and B). AD rats exhibited increased GFAP reaction (*p* < 0.0001) compared to control (Fig. 8C), which was attenuated in AD rats treated with metformin (*p* < 0.0001) compared to AD rats (Fig. 8D). Similarly, calretinin showed strong immunoreactivity in normal and metformin-treated groups (Fig. 9A and B), decreased in the AD group (*p* < 0.0001), and then increased after metformin treatment (*p* = 0.0019) compared to AD rats (Fig. 9C and D).

## Discussion

Alzheimer’s disease (AD) is a progressively growing concern, with its inextricable link to aging presenting a formidable challenge (Tzioras et al. 2023). Consequently, addressing AD effectively requires a thorough understanding of its mechanistic underpinnings and their connections to related disorders, to identify viable therapeutic avenues. This study delved into the potential role of metformin in treating AD and sought to establish a connection between AD pathogenesis and insulin resistance within the brain. According to the outcomes of the existing study, metformin, a medication commonly prescribed for diabetes, has the potential to enhance cognitive function, alleviate stress, improve neurotransmitter activity, and enhance insulin pathways within the brain. The possible outcome of this could result in beneficial effects for alleviating symptoms of Alzheimer’s disease.

Additionally, the study’s findings shed light on the multifaceted impact of AD induction through D-gal and AlCl_3_, which not only manifested in cognitive decline but also encompassed physical alterations such as weight loss in the rats. A discernible trend towards memory impairment was observed, alongside elevated glucose levels and diminished brain weight. It was discerned that AD induction exerted a detrimental influence on the frontal memory region, potentially causing neuronal damage and concomitant reductions in brain glucose levels and energy metabolism. These observations resonated with the work of Nitsch et al., who demonstrated the interplay between glucose, energy levels, and cognitive faculties (Nitsch and Hoyer 1991). Moreover, prior studies have established correlations between AD induction, fluctuations in glucose levels, and cognitive dysfunction (Amin et al. 2013; Mostafa et al. 2016; Tanokashira et al. 2018).

Notably, the study illuminated that AD’s impact extended beyond cognitive impairment, impacting crucial organs and systems, including liver and kidney function alterations driven by lipid and ion imbalances. The observed increase in liver function was in line with previous research associating liver function, particularly non-alcoholic liver disease, with heightened dementia risk (Solfrizzi et al. 2020; Shang et al. 2022). Similarly, a study in chronic schizophrenia highlighted increased C-reactive protein, an indicator of liver response to endotoxins (Liu et al. 2022). A former study also corroborated prior evidence linking cognitive impairment with end-stage renal disease and severity-scaled cognitive decline in chronic kidney disease (Murray et al. 2006).

Crucially, the study identified elevated glucose, HbA1C, and HOMA-IR levels as markers of insulin resistance. This phenomenon has been linked to diminished glucose metabolism in the brain and increased Aβ deposition. Impaired insulin signaling is a hallmark of AD, prompting some to label it “type 3 diabetes” due to its ramifications on memory and cognitive function (Wakabayashi et al. 2019). Encouragingly, metformin administration effectively ameliorated blood sugar control, HOMA-IR, and HbA1c in AD rats. These findings aligned with existing literature (Derosa et al. 2017; Liu et al. 2022).

Furthermore, AD’s impact on the insulin signaling pathway manifested in heightened hepatic glucose and lipid production, culminating in lipotoxicity (Kelley et al. 2003; Bugianesi et al. 2005). Noteworthy alterations in free fatty acid levels, apoB decomposition, hypertriglyceridemia, and HDL levels also occurred (Ormazabal et al. 2018) Metformin treatment effectively mitigated total cholesterol, LDL, and triglyceride levels (Gillani et al. 2021). The study underscored the pivotal roles of oxidative stress and neuroinflammation in AD pathogenesis (Li et al. 2023). Previous experiments have attested to oxidative stress’s contribution to AD onset through Aβ-mediated mechanisms, inducing mitochondrial dysfunction, glucose metabolism disruption, synaptic plasticity diminishing, distorted signal transduction, neuronal injury, and progressive neurodegeneration (Butterfield and Boyd-Kimball 2019; Hampel et al. 2021). Consistent with these findings, chemically induced AD displayed elevated inflammatory indicators and oxidative stress, alongside weakened antioxidant systems (Kazmi et al. 2023). Metformin effectively reduced hippocampal oxidative stress and enhanced antioxidant defenses, aligning with similar research demonstrating metformin’s capacity to lower malondialdehyde levels and elevate total antioxidant capacity (Gorgich et al. 2021).

The elevation of oxidative stress paralleled heightened hippocampal iron and zinc levels, potentially contributing to free radical generation via Fenton reactions, a phenomenon implicated in neurodegenerative (Gozzelino and Arosio 2016). These findings corroborated earlier studies indicating elevated ion levels, including iron, zinc, and copper, in sporadic AD (Lovell et al. 1998). Notably, metformin’s ameliorative effects on these anomalies resonated with prior reports (Mascitelli and Pezzetta 2008; Mustafa et al. 2022).

Neurotransmitter assessment illuminated notable declines in dopamine and acetylcholine levels, coupled with heightened acetylcholine esterase activity, a pattern ameliorated by metformin treatment. These findings concurred with studies showcasing metformin’s potential to rectify synaptic deficits, enhance memory, and improve cognition in AD animal models (Pilipenko et al. 2020; Saffari et al. 2020; Thinnes et al. 2021).

Consequently, the study demonstrated that metformin effectively reduced Aβ levels. These findings mirrored previous investigations wherein metformin attenuated hippocampal Aβ1–42 levels (Li et al. 2012). Correspondingly, metformin’s potential to ameliorate microglial autophagy impairment and mitigate Aβ load and NP tau pathology was consonant with Chen et al.‘s findings (Chen et al. 2021). The study further scrutinized IRS-2, a key component of insulin signaling in the brain. Notably, sporadic AD displayed diminished IRS-2 levels, a deficit ameliorated by metformin treatment. These results harmonized with studies demonstrating metformin-induced IRS-2 upregulation (Rice et al. 2011).

Early pathology and immunohistopathology of hippocampal were investigated in the current study. Histopathological examination unveiled neuronal damage and degeneration, accompanied by robust astrogliosis (GFAP immunoreactivity) and attenuated calretinin staining in AD rats. Encouragingly, metformin mitigated these adverse effects, in line with prior research highlighting metformin’s potential to ameliorate hippocampal microgliosis (Pilipenko et al. 2020). The decrease in GFAP expression resonated with Miguel-Hidalgo’s findings, indicating the neuroprotective power of memantine on β-amyloid-induced rat models, and concluded that memantine-treated animals had noteworthy falls in GFAP immunostaining (Miguel-Hidalgo et al. 2002).

In conclusion, this study revealed the intertwining nature of AD and insulin signaling, emphasizing their interconnectedness rather than treating them as isolated phenomena. Importantly, metformin emerges as an encouraging therapeutic agent for addressing AD, shedding light on its potential to mitigate cognitive decline and tackle the intricate mechanisms.

## Electronic Supplementary Material

Below is the link to the electronic supplementary material.

Supplementary Material 1 (DOCX 631 KB)

## Data Availability

All data that support the current study are available in the manuscript and supporting data file.
