# Supplementary material for: Metformin effectively alleviates the symptoms of Alzheimer in rats by lowering amyloid β deposition and enhancing the insulin signal
Source: Metab Brain Dis. 2024 Nov 25;40(1):41. doi: 10.1007/s11011-024-01422-8 (PMC11588952; doi:10.1007/s11011-024-01422-8)
Supplement: Supplementary file 1 — Supplementary Material 1 (DOCX 631 KB) [file 11011_2024_1422_MOESM1_ESM.pdf]

Supplementary information

Metformin effectively alleviates the symptoms of Alzheimer in rats by lowering amyloid β deposition and enhancing the insulin signal

Hamed A. Abosharaf[1*], Yasmin Elsonbaty[1], Ehab Tousson[2], Tarek M. Mohamed[1*]

[1] Biochemistry Division, Chemistry Department, Faculty of Science, Tanta University, Tanta 31527, Egypt

[2] Zoology Department, Faculty of Science, Tanta University, Tanta, 31527, Egypt

*Authors to whom correspondence should be addressed

Hamed A. Abosharaf
Email: hamed_biochemistry@science.tanta.edu.eg
Tel: 01030316872

Tarek M. Mohamed
Email: tarek.ali@science.tanta.edu.eg
Tel.: 01006338769

Methods:

Quantification of the oxidative/ antioxidative parameters

Measuring of MDA level

In a nutshell, 2.5 mL of TCA and 0.5 mL of 10% of the tissue homogenates were mixed before the homogenates were centrifuged at 3,000 rpm for 10 min. After that, 1 mL of TBA and 2 mL of the supernatant were combined, and the mixture was quickly cooled after being heated for 15 minutes. Following the addition of 4 mL of n-butyl alcohol, the tubes were shaken, centrifuged at 3,000 rpm for 10 min, and the absorbance at 532 nm was measured. Using the value of 532 = 156 $mM^{-1}cm^{-1}$, the MDA level was presented as nmol/mg of protein.

Measuring of GSH level

In a sentence, 250 µL of 40% DTNB and 50 µL of the tissue homogenates were combined with DTNB buffer (0.1 M sodium phosphate and 1 mM EDTA, pH 7.5). At 412 nm, the absorbance

was subsequently determined. Using a standard curve, the GSH concentration was estimated as nmol/mg of protein.

Statistical analysis:

The sample size (n) in the study was determined using the following equation:

$$n = \frac{\frac{z^2 \cdot P(1-P)}{e^2}}{1 + \left(\frac{z^2 \cdot P}{e^2 N}(1-P)\right)}$$

Where Z is the critical value corresponding to level of confidence (95%), P is population proportion (0.5), e is margin of error (0.04), and N population size.

Results:

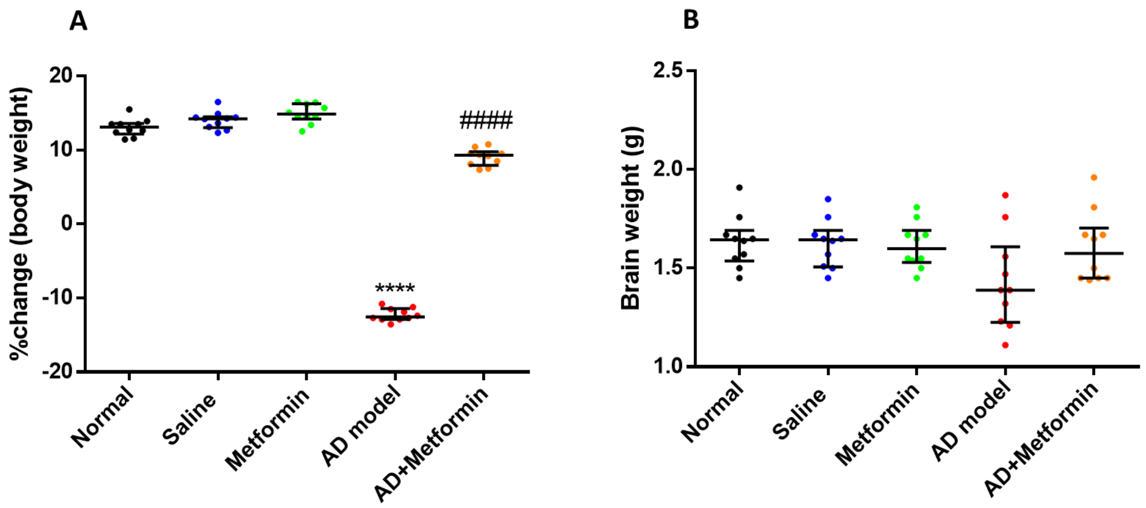

Fig. S1. The impact of metformin on the change in the body weight (A) and the brain weight (B) in AD- rat model. The results were presented as mean ± SD, (n=10), P<0.05 was counted a significant. $^{*}P$, $^{\#}P$; the significancy against normal and AD model respectively.

Table S1. Influence of metformin on serum liver enzymes (ALT and AST), kidney function biomarkers (urea and creatinine), Lipid profile (cholesterol, triacylglycerol, LDL, and HDL), iron, and zinc levels in all treated rats

Serum Parameters	Rat's groups				
	G1	G2	G3	G4	G5
ALT (IU)	35.13±3.3	33.93±4.15	35.8±3.15	66.3±2.6[a]	43.66±2.1[a,b]
AST (IU)	41.8±3.13	43.8±3.2	45.36±3.3	95.2±2.3[a]	66.63±1.4[a,b]
Urea (mg/dl)	24.16±3.28	25.13±4.3	23.63±5.5	56.6±1.12[a]	34.2±1.1[a,b]
Creatinine (mg/dl)	0.76±0.098	0.67±0.28	0.77±0.12	2.7±0.1[a]	1.3±0.1[a,b]
TG (mg/dl))	45.4±4.06	48.1±3.6	49.1±6.6	151.1±2.4[a]	73.06±2.9[a,b]
Cholesterol (mg/dl)	90.3±4.1	90.4±4.7	92.5±1.3	208.1±2.9[a]	133.1±2.72[a,b]
HDL (mg/dl)	54.4±3.14	55.7±5.9	56.8±6.8	31.3±3.9[a]	41.97±2.1[a,b]
LDL (mg/dl)	27.7±2.3	25.1±1.6	26.17±2.1	146.5±1.31[a]	79.8±7.6[a,b]
Iron (µmol/L)	0.7±0.2	0.71±0.17	0.63±0.17	2.6±0.15[a]	1.17±0.02[a,b]
Zinc (mg/dl)	59.1±3.8	56.43±5.5	58.03±2.8	95.56±1.5[a]	62.36±2.25[a,b]

The data are shown as the mean ± SD (n =10). a and b are the significancy versus control (G1) and AD-rat model (G4) respectively. $P < 0.05$ was count a significant.

Normality test of arrival time results

	Col. stats	A	B	C	D	E
		Normal	Saline	Metformin	AD model	AD+Metformin
		Y	Y	Y	Y	Y
1	Number of values	10	10	10	10	10
2						
3						
4	Mean	50.60	60.80	61.10	163.5	87.80
5	Std. Deviation	3.026	8.121	3.479	5.681	8.991
6	Std. Error of Mean	0.9568	2.568	1.100	1.797	2.843
7						
8	D'Agostino & Pearson omnibus normality test					
9	K2	1.479	2.671	0.02261	1.411	0.5990
10	P value	0.4774	0.2630	0.9888	0.4937	0.7412
11	Passed normality test (alpha=0.05)?	Yes	Yes	Yes	Yes	Yes
12	P value summary	ns	ns	ns	ns	ns
13						
14	Shapiro-Wilk normality test					
15	W	0.9105	0.8949	0.9815	0.9313	0.9582
16	P value	0.2843	0.1922	0.9727	0.4610	0.7650
17	Passed normality test (alpha=0.05)?	Yes	Yes	Yes	Yes	Yes
18	P value summary	ns	ns	ns	ns	ns

Normality test of HbA1c results

	Col. stats	A	B	C	D	E
		Normal	Saline	Metformin	AD model	AD+Metformin
		Y	Y	Y	Y	Y
1	Number of values	10	10	10	10	10
2						
3						
4	Mean	4.117	4.037	4.168	8.635	6.312
5	Std. Deviation	0.3256	0.3192	0.4188	0.6201	0.7521
6	Std. Error of Mean	0.1030	0.1010	0.1324	0.1961	0.2378
7						
8	D'Agostino & Pearson omnibus normality test					
9	K2	1.395	1.165	1.974	0.3256	0.1010
10	P value	0.4978	0.5585	0.3727	0.8498	0.9507
11	Passed normality test (alpha=0.05)?	Yes	Yes	Yes	Yes	Yes
12	P value summary	ns	ns	ns	ns	ns
13						
14	Shapiro-Wilk normality test					
15	W	0.9110	0.8915	0.9127	0.9251	0.9490
16	P value	0.2878	0.1763	0.2998	0.4017	0.6572
17	Passed normality test (alpha=0.05)?	Yes	Yes	Yes	Yes	Yes
18	P value summary	ns	ns	ns	ns	ns

Normality test of glucose results

	Col. stats	A	B	C	D	E
		Normal	Saline	Metformin	AD model	AD+Metformin
		Y	Y	Y	Y	Y
1	Number of values	10	10	10	10	10
2						
3						
4	Mean	83.21	87.89	85.19	218.2	121.1
5	Std. Deviation	7.089	8.080	7.527	12.18	5.496
6	Std. Error of Mean	2.242	2.555	2.380	3.852	1.738
7						
8	D'Agostino & Pearson omnibus normality test					
9	K2	0.4898	2.057	1.078	3.130	1.020
10	P value	0.7828	0.3575	0.5832	0.2091	0.6004
11	Passed normality test (alpha=0.05)?	Yes	Yes	Yes	Yes	Yes
12	P value summary	ns	ns	ns	ns	ns
13						
14	Shapiro-Wilk normality test					
15	W	0.9175	0.8033	0.9216	0.9069	0.9556
16	P value	0.3363	0.0159	0.3707	0.2601	0.7346
17	Passed normality test (alpha=0.05)?	Yes	No	Yes	Yes	Yes
18	P value summary	ns	*	ns	ns	ns

Normality test of insulin results

	Col. stats	A	B	C	D	E
		Normal	Saline	Metformin	AD model	AD+Metformin
		Y	Y	Y	Y	Y
1	Number of values	10	10	10	10	10
2						
3						
4	Mean	0.4452	0.4545	0.4819	2.251	1.195
5	Std. Deviation	0.07456	0.08772	0.08441	0.2875	0.1002
6	Std. Error of Mean	0.02358	0.02774	0.02669	0.09090	0.03170
7						
8	D'Agostino & Pearson omnibus normality test					
9	K2	0.2165	0.5267	2.113	7.145	2.146
10	P value	0.8974	0.7685	0.3477	0.0281	0.3419
11	Passed normality test (alpha=0.05)?	Yes	Yes	Yes	No	Yes
12	P value summary	ns	ns	ns	*	ns
13						
14	Shapiro-Wilk normality test					
15	W	0.9596	0.9628	0.9314	0.8901	0.9259
16	P value	0.7812	0.8173	0.4614	0.1699	0.4086
17	Passed normality test (alpha=0.05)?	Yes	Yes	Yes	Yes	Yes
18	P value summary	ns	ns	ns	ns	ns

Normality test of HOMA-IR results

	Col. stats	A	B	C	D	E
		Normal	Saline	Metformin	AD model	AD+Metformin
		Y	Y	Y	Y	Y
1	Number of values	10	10	10	10	10
2						
3						
4	Mean	0.06795	0.0835	0.06129	1.224	0.2920
5	Std. Deviation	0.01771	0.02405	0.02458	0.1323	0.08483
6	Std. Error of Mean	0.005599	0.007606	0.007774	0.04185	0.02682
7						
8	D'Agostino & Pearson omnibus normality test					
9	K2	3.790	0.3439	9.325	0.01757	0.7834
10	P value	0.1503	0.8420	0.0094	0.9913	0.6759
11	Passed normality test (alpha=0.05)?	Yes	Yes	No	Yes	Yes
12	P value summary	ns	ns	**	ns	ns
13						
14	Shapiro-Wilk normality test					
15	W	0.8758	0.9635	0.8515	0.9857	0.9431
16	P value	0.1166	0.8247	0.0605	0.9883	0.5874
17	Passed normality test (alpha=0.05)?	Yes	Yes	Yes	Yes	Yes
18	P value summary	ns	ns	ns	ns	ns
19						

Normality test of Zinc results

Col. stats	A	B	C	D	E
	Normal	Saline	Metformin	AD model	AD+Metformin
	Y	Y	Y	Y	Y
1 Number of values	10	10	10	10	10
2					
3					
4 Mean	0.9090	0.8407	0.8363	1.957	1.415
5 Std. Deviation	0.1493	0.1595	0.1558	0.1916	0.1579
6 Std. Error of Mean	0.04720	0.05042	0.04927	0.06059	0.04994
7					
8 D'Agostino & Pearson omnibus normality test					
9 K2	0.2200	0.3355	3.666	0.04861	0.4101
10 P value	0.8958	0.8456	0.1599	0.9760	0.8146
11 Passed normality test (alpha=0.05)?	Yes	Yes	Yes	Yes	Yes
12 P value summary	ns	ns	ns	ns	ns
13					
14 Shapiro-Wilk normality test					
15 W	0.9564	0.9667	0.9197	0.9711	0.9715
16 P value	0.7447	0.8587	0.3542	0.9009	0.9042
17 Passed normality test (alpha=0.05)?	Yes	Yes	Yes	Yes	Yes
18 P value summary	ns	ns	ns	ns	ns
19					

Normality test of Iron results

Col. stats	A	B	C	D	E
	Normal	Saline	Metformin	AD model	AD+Metformin
	Y	Y	Y	Y	Y
1 Number of values	10	10	10	10	10
2					
3					
4 Mean	24.21	21.30	22.41	37.90	28.47
5 Std. Deviation	1.869	2.101	1.884	2.832	3.054
6 Std. Error of Mean	0.5909	0.6645	0.5959	0.8956	0.9658
7					
8 D'Agostino & Pearson omnibus normality test					
9 K2	0.4916	0.8106	1.769	1.866	2.573
10 P value	0.7821	0.6668	0.4128	0.3935	0.2762
11 Passed normality test (alpha=0.05)?	Yes	Yes	Yes	Yes	Yes
12 P value summary	ns	ns	ns	ns	ns
13					
14 Shapiro-Wilk normality test					
15 W	0.9834	0.9771	0.8860	0.9014	0.8320
16 P value	0.9807	0.9481	0.1526	0.2268	0.0354
17 Passed normality test (alpha=0.05)?	Yes	Yes	Yes	Yes	No
18 P value summary	ns	ns	ns	ns	*

Normality test of Dopamine results

Col. stats	A	B	C	D	E
	Normal	Saline	Metformin	AD model	AD+Metformin
	Y	Y	Y	Y	Y
1 Number of values	10	10	10	10	10
2					
3					
4 Mean	2.966	2.881	2.812	1.036	1.617
5 Std. Deviation	0.1682	0.2903	0.3770	0.1604	0.4593
6 Std. Error of Mean	0.05319	0.09179	0.1192	0.05071	0.1452
7					
8 D'Agostino & Pearson omnibus normality test					
9 K2	0.5635	7.537	7.529	2.884	3.120
10 P value	0.7545	0.0231	0.0232	0.2364	0.2101
11 Passed normality test (alpha=0.05)?	Yes	No	No	Yes	Yes
12 P value summary	ns	*	*	ns	ns
13					
14 Shapiro-Wilk normality test					
15 W	0.9608	0.8907	0.8524	0.8939	0.8163
16 P value	0.7953	0.1726	0.0620	0.1875	0.0228
17 Passed normality test (alpha=0.05)?	Yes	Yes	Yes	Yes	No
18 P value summary	ns	ns	ns	ns	*

Normality test of Acetylcholine results

Col. stats	A	B	C	D	E
	Normal	Saline	Metformin	AD model	AD+Metformin
	Y	Y	Y	Y	Y
4 Mean	64.86	63.12	63.27	15.60	23.35
5 Std. Deviation	3.964	1.337	3.566	2.582	3.297
6 Std. Error of Mean	1.253	0.4226	1.128	0.8165	1.043
7					
8 D'Agostino & Pearson omnibus normality test					
9 K2	3.605	0.3136	4.016	1.332	0.5500
10 P value	0.1649	0.8549	0.1343	0.5137	0.7596
11 Passed normality test (alpha=0.05)?	Yes	Yes	Yes	Yes	Yes
12 P value summary	ns	ns	ns	ns	ns
13					
14 Shapiro-Wilk normality test					
15 W	0.8263	0.9799	0.8878	0.9548	0.9342
16 P value	0.0302	0.9645	0.1600	0.7254	0.4900
17 Passed normality test (alpha=0.05)?	No	Yes	Yes	Yes	Yes
18 P value summary	*	ns	ns	ns	ns

Normality test of Acetylcholine esterase

	Col. stats	A	B	C	D	E
		Normal	Saline	Metformin	AD model	AD+Metformin
		Y	Y	Y	Y	Y
4	Mean	0.9080	0.8806	0.9274	2.217	1.954
5	Std. Deviation	0.1502	0.07822	0.09376	0.2078	0.4066
6	Std. Error of Mean	0.04748	0.02474	0.02965	0.06571	0.1286
7						
8	D'Agostino & Pearson omnibus normality test					
9	K2	14.53	0.3579	4.822	1.173	1.625
10	P value	0.0007	0.8361	0.0897	0.5562	0.4438
11	Passed normality test (alpha=0.05)?	No	Yes	Yes	Yes	Yes
12	P value summary	***	ns	ns	ns	ns
13						
14	Shapiro-Wilk normality test					
15	W	0.7807	0.9385	0.9068	0.9021	0.9028
16	P value	0.0084	0.5370	0.2598	0.2309	0.2353
17	Passed normality test (alpha=0.05)?	No	Yes	Yes	Yes	Yes
18	P value summary	**	ns	ns	ns	ns
19						
20	KS normality test					
21	KS distance	0.2477	0.1461	0.1719	0.2552	0.2291
22	P value	0.0827	0.2000	0.2000	0.0639	0.1500
23	Passed normality test (alpha=0.05)?	Yes	Yes	Yes	Yes	Yes
24	P value summary	ns	ns	ns	ns	ns

Normality test of MDA results

	Col. stats	A	B	C	D	E
		Normal	Saline	Metformin	AD model	AD+Metformin
		Y	Y	Y	Y	Y
4	Mean	0.3728	0.3981	0.4106	1.549	0.7022
5	Std. Deviation	0.05224	0.1106	0.08114	0.1856	0.1729
6	Std. Error of Mean	0.01652	0.03496	0.02566	0.05870	0.05468
7						
8	D'Agostino & Pearson omnibus normality test					
9	K2	0.3372	1.039	3.351	1.624	0.7498
10	P value	0.8449	0.5947	0.1873	0.4440	0.6874
11	Passed normality test (alpha=0.05)?	Yes	Yes	Yes	Yes	Yes
12	P value summary	ns	ns	ns	ns	ns
13						
14	Shapiro-Wilk normality test					
15	W	0.9493	0.9258	0.8580	0.8876	0.9451
16	P value	0.6601	0.4076	0.0722	0.1595	0.6114
17	Passed normality test (alpha=0.05)?	Yes	Yes	Yes	Yes	Yes
18	P value summary	ns	ns	ns	ns	ns
19						
20	KS normality test					
21	KS distance	0.2059	0.2135	0.2724	0.2245	0.1309
22	P value	0.2000	0.2000	0.0341	0.1724	0.2000
23	Passed normality test (alpha=0.05)?	Yes	Yes	No	Yes	Yes
24	P value summary	ns	ns	*	ns	ns

Normality test of GSH results

	Col. stats	A	B	C	D	E
		Normal	Saline	Metformin	AD model	AD+Metformin
		Y	Y	Y	Y	Y
3						
4	Mean	1.855	1.809	2.005	0.6729	1.028
5	Std. Deviation	0.2517	0.2400	0.1761	0.1621	0.2556
6	Std. Error of Mean	0.07960	0.07590	0.05568	0.05127	0.08084
7						
8	D'Agostino & Pearson omnibus normality test					
9	K2	0.2381	0.2062	0.2301	1.397	0.4878
10	P value	0.8878	0.9020	0.8913	0.4973	0.7835
11	Passed normality test (alpha=0.05)?	Yes	Yes	Yes	Yes	Yes
12	P value summary	ns	ns	ns	ns	ns
13						
14	Shapiro-Wilk normality test					
15	W	0.9752	0.9618	0.9490	0.9349	0.9383
16	P value	0.9347	0.8061	0.6563	0.4979	0.5343
17	Passed normality test (alpha=0.05)?	Yes	Yes	Yes	Yes	Yes
18	P value summary	ns	ns	ns	ns	ns
19						
20	KS normality test					
21	KS distance	0.1143	0.1353	0.2113	0.1569	0.1349
22	P value	0.2000	0.2000	0.2000	0.2000	0.2000
23	Passed normality test (alpha=0.05)?	Yes	Yes	Yes	Yes	Yes
24	P value summary	ns	ns	ns	ns	ns

Normality test of SOD results

	Col. stats	A	B	C	D	E
		Normal	Saline	Metformin	AD model	AD+Metformin
		Y	Y	Y	Y	Y
1	Number of values	10	10	10	10	10
2						
3						
4	Mean	2.586	2.613	2.668	0.7406	1.207
5	Std. Deviation	0.4202	0.3044	0.3902	0.2280	0.4223
6	Std. Error of Mean	0.1329	0.09626	0.1234	0.07209	0.1335
7						
8	D'Agostino & Pearson omnibus normality test					
9	K2	3.088	0.6432	2.594	1.592	1.068
10	P value	0.2135	0.7250	0.2733	0.4510	0.5862
11	Passed normality test (alpha=0.05)?	Yes	Yes	Yes	Yes	Yes
12	P value summary	ns	ns	ns	ns	ns
13						
14	Shapiro-Wilk normality test					
15	W	0.8277	0.9516	0.7735	0.8949	0.9492
16	P value	0.0314	0.6880	0.0069	0.1925	0.6590
17	Passed normality test (alpha=0.05)?	No	Yes	No	Yes	Yes
18	P value summary	*	ns	**	ns	ns
19						
20	KS normality test					
21	KS distance	0.2430	0.1186	0.3153	0.2216	0.1805
22	P value	0.0968	0.2000	0.0056	0.1876	0.2000
23	Passed normality test (alpha=0.05)?	Yes	Yes	No	Yes	Yes
24	P value summary	ns	ns	**	ns	ns

Normality test of GPx results

	Col. stats	A	B	C	D	E
		Normal	Saline	Metformin	AD model	AD+Metformin
		Y	Y	Y	Y	Y
1	Number of values	10	10	10	10	10
2						
3						
4	Mean	21.15	21.24	22.22	12.50	18.65
5	Std. Deviation	2.087	2.481	2.292	2.584	2.304
6	Std. Error of Mean	0.6599	0.7847	0.7247	0.8170	0.7287
7						
8	D'Agostino & Pearson omnibus normality test					
9	K2	0.6367	0.4218	7.213	0.5583	2.185
10	P value	0.7274	0.8099	0.0271	0.7564	0.3353
11	Passed normality test (alpha=0.05)?	Yes	Yes	No	Yes	Yes
12	P value summary	ns	ns	*	ns	ns
13						
14	Shapiro-Wilk normality test					
15	W	0.9576	0.9915	0.8461	0.9752	0.9613
16	P value	0.7581	0.9983	0.0522	0.9347	0.8004
17	Passed normality test (alpha=0.05)?	Yes	Yes	Yes	Yes	Yes
18	P value summary	ns	ns	ns	ns	ns
19						
20	KS normality test					
21	KS distance	0.1456	0.1029	0.2939	0.1073	0.1611
22	P value	0.2000	0.2000	0.0144	0.2000	0.2000
23	Passed normality test (alpha=0.05)?	Yes	Yes	No	Yes	Yes
24	P value summary	ns	ns	*	ns	ns

Normality test of Catalase

Col. stats	A	B	C	D	E
	Normal	Saline	Metformin	AD model	AD+Metformin
	Y	Y	Y	Y	Y
1 Number of values	10	10	10	10	10
2					
3					
4 Mean	21.15	21.24	22.26	12.70	18.65
5 Std. Deviation	2.087	2.481	2.244	2.618	2.304
6 Std. Error of Mean	0.6599	0.7847	0.7096	0.8278	0.7287
7					
8 D'Agostino & Pearson omnibus normality test					
9 K2	0.6367	0.4218	8.196	1.153	2.185
10 P value	0.7274	0.8099	0.0166	0.5619	0.3353
11 Passed normality test (alpha=0.05)?	Yes	Yes	No	Yes	Yes
12 P value summary	ns	ns	*	ns	ns
13					
14 Shapiro-Wilk normality test					
15 W	0.9576	0.9915	0.8462	0.9663	0.9613
16 P value	0.7581	0.9983	0.0524	0.8546	0.8004
17 Passed normality test (alpha=0.05)?	Yes	Yes	Yes	Yes	Yes
18 P value summary	ns	ns	ns	ns	ns
19					
20 KS normality test					
21 KS distance	0.1456	0.1029	0.2985	0.1199	0.1611
22 P value	0.2000	0.2000	0.0118	0.2000	0.2000
23 Passed normality test (alpha=0.05)?	Yes	Yes	No	Yes	Yes
24 P value summary	ns	ns	*	ns	ns

ANOVA tables for all obtained statistical measurements.

Arrival time:

ANOVA table	SS	DF	MS	F (DFn, DFd)	P value
Treatment (between columns)	85100	4	21275	F (4, 45) = 531.0	P < 0.0001
Residual (within columns)	1803	45	40.07		
Total	86903	49			

HbA1c level:

ANOVA table	SS	DF	MS	F (DFn, DFd)	P value
Treatment (between columns)	163.0	4	40.76	F (4, 45) = 152.8	P < 0.0001
Residual (within columns)	12.00	45	0.2667		
Total	175.0	49			

Glucose level:

ANOVA table	SS	DF	MS	F (DFn, DFd)	P value
Treatment (between columns)	132466	4	33116	F (4, 45) = 472.0	P < 0.0001
Residual (within columns)	3157	45	70.16		
Total	135623	49			

Insulin level:

ANOVA table	SS	DF	MS	F (DFn, DFd)	P value
Treatment (between columns)	24.71	4	6.177	F (4, 45) = 273.2	P < 0.0001
Residual (within columns)	1.018	45	0.02261		
Total	25.73	49			

HOMA-IR:

ANOVA table	SS	DF	MS	F (DFn, DFd)	P value
Treatment (between columns)	10.01	4	2.503	F (4, 45) = 477.5	P < 0.0001
Residual (within columns)	0.2359	45	0.005241		
Total	10.25	49			

Zinc level:

ANOVA table	SS	DF	MS	F (DFn, DFd)	P value
Treatment (between columns)	9.650	4	2.412	F (4, 45) = 90.27	P < 0.0001
Residual (within columns)	1.203	45	0.02673		
Total	10.85	49			

Iron level:

ANOVA table	SS	DF	MS	F (DFn, DFd)	P value
Treatment (between columns)	1823	4	455.7	F (4, 45) = 79.11	P < 0.0001
Residual (within columns)	259.3	45	5.761		
Total	2082	49			

Dopamine level:

ANOVA table	SS	DF	MS	F (DFn, DFd)	P value
Treatment (between columns)	31.00	4	7.751	F (4, 45) = 78.87	P < 0.0001
Residual (within columns)	4.422	45	0.09827		
Total	35.43	49			

Acetylcholine level:

ANOVA table	SS	DF	MS	F (DFn, DFd)	P value
Treatment (between columns)	23845	4	5961	F (4, 45) = 624.2	P < 0.0001
Residual (within columns)	429.8	45	9.550		
Total	24274	49			

AchE level:

ANOVA table	SS	DF	MS	F (DFn, DFd)	P value
Treatment (between columns)	17.07	4	4.268	F (4, 45) = 86.75	P < 0.0001
Residual (within columns)	2.214	45	0.04919		
Total	19.28	49			

MDA level:

ANOVA table	SS	DF	MS	F (DFn, DFd)	P value
Treatment (between columns)	10.01	4	2.503	F (4, 45) = 145.7	P < 0.0001
Residual (within columns)	0.7730	45	0.01718		
Total	10.78	49			

GSH level:

ANOVA table	SS	DF	MS	F (DFn, DFd)	P value
Treatment (between columns)	13.80	4	3.451	F (4, 45) = 70.83	P < 0.0001
Residual (within columns)	2.192	45	0.04872		
Total	16.00	49			

SOD level:

ANOVA table	SS	DF	MS	F (DFn, DFd)	P value
Treatment (between columns)	33.74	4	8.434	F (4, 45) = 64.70	P < 0.0001
Residual (within columns)	5.867	45	0.1304		
Total	39.60	49			

GPx level:

ANOVA table	SS	DF	MS	F (DFn, DFd)	P value
Treatment (between columns)	622.9	4	155.7	F (4, 45) = 28.06	P < 0.0001
Residual (within columns)	249.7	45	5.550		
Total	872.7	49			

Catalase level:

ANOVA table	SS	DF	MS	F (DFn, DFd)	P value
Treatment (between columns)	622.9	4	155.7	F (4, 45) = 28.06	P < 0.0001
Residual (within columns)	249.7	45	5.550		
Total	872.7	49			
